# A comparison of epidemiology and clinical outcomes between influenza A H1N1pdm09 and H3N2 based on multicenter surveillance from 2014 to 2018 in South Korea

**DOI:** 10.1111/irv.12795

**Published:** 2020-08-25

**Authors:** Jin Gu Yoon, Ji Yun Noh, Won Suk Choi, Jacob Lee, Jin Soo Lee, Seong‐Heon Wie, Young Keun Kim, Hye Won Jeong, Shin Woo Kim, Kyung‐Hwa Park, Joon Young Song, Hee Jin Cheong, Woo Joo Kim

**Affiliations:** ^1^ Division of Infectious Diseases Department of Internal Medicine Korea University College of Medicine Seoul Korea; ^2^ Asian Pacific Influenza Institute (APII) Seoul Korea; ^3^ Division of Infectious Diseases Department of Internal Medicine Kangnam Sacred Heart Hospital Hallym University College of Medicine Seoul Korea; ^4^ Division of Infectious Diseases Department of Internal Medicine Inha University School of Medicine Incheon Korea; ^5^ Division of Infectious Diseases Department of Internal Medicine The Catholic University of Korea College of Medicine St. Vincent's Hospital Suwon Korea; ^6^ Division of Infectious Diseases Department of Internal Medicine Yonsei University Wonju College of Medicine Wonju Korea; ^7^ Division of Infectious Diseases Department of Internal Medicine Chungbuk National University College of Medicine Cheongju Korea; ^8^ Division of Infectious Diseases Department of Internal Medicine Kyungpook National University School of Medicine Daegu Korea; ^9^ Division of Infectious Diseases Chonnam National University Medical School Gwangju Korea

**Keywords:** epidemiology, H1N1 Subtype, H3N2 subtype, hospital mortality, influenza A virus, pneumonia

## Abstract

**Background:**

After pandemic, A(H1N1)pdm09 is generally known to be associated with younger adults' infection and greater severity than seasonal A(H3N2) but some inconsistences between recent studies exist.

**Objectives:**

We aimed to compare the epidemiology and clinical outcomes of A(H1N1)pdm09 and A(H3N2) to verify and consolidate about the knowledge of known differences of subtypes.

**Methods:**

Data were retrospectively collected from the hospital‐based influenza morbidity and mortality surveillance in South Korea in nine tertiary care hospitals, from August 31, 2014, to August 25, 2018. Patients with H1N1pdm09 or H3N2 infection admitted in the emergency room or ward were recruited.

**Results:**

A total of 1747 patients had influenza A and were divided into two groups those with A(H1N1)pdm09 (n = 240) and those with A(H3N2) (n = 1507). A(H1N1)pdm09 group had younger age (mean age ± standard deviation 50.0 ± 18.8 in H1N1 vs 53.4 ± 21.1 in H3N2, *P* = .030), lower influenza vaccination (27.9% vs 43.9%, *P* < .001) and pneumococcal vaccination rates (41.0% vs 51.9%, *P* < .001), and fewer underlying diseases (67.5% vs 74.0%, *P* = .035) than the A(H3N2) group. Influenza A subtypes were not associated with pneumonia risk (adjusted odds ratios [AOR] of A(H1N1)pdm09: 0.7 [95% confidence interval [CI]: 0.4‐1.2, *P* = .172]) and in‐hospital mortality (hazard ratio (HR) of A(H1N1)pdm09: 1.0 (95% CI: 0.3‐3.1, *P* = .983)). Influenza vaccination reduced in‐hospital mortality in hospitalized patients (HR: 0.3 (95% CI: 0.1‐0.7), *P* = .005).

**Conclusions:**

A(H1N1)pdm09 infection was more common in younger patients without significant difference in pneumonia risk and in‐hospital mortality between subtypes. Influenza vaccination was associated with reduced in‐hospital mortality.

## 1 BACKGROUND

Influenza virus infection causes acute respiratory illness, which is accompanied by fever, general weakness, myalgia, and occasionally serious life‐threatening illness or death. Major influenza viruses that cause seasonal epidemics worldwide are influenza A and influenza B. Influenza A viruses have specific subtypes, which depend upon the type of surface antigens as follows: hemagglutinins (H) and neuraminidases (N). In 2009, a novel influenza A H1N1 strain emerged and circulated throughout the world, which was named A(H1N1)pdm09. Currently, it is now one of the most important seasonal influenza A strains along with H3N2. Exact forecasting of dominant influenza type is very important for global vaccination strategies. However, it is unpredictable which influenza type will prevail year by year and varies from country to country. Influenza surveillance in South Korea showed that A(H1N1)pdm09 subtype was more frequent than A(H3N2) in 2015‐2016 (H1N1 44.1%; H3N2 4.7%; B 51.1%). In contrast, A(H3N2) subtype was more frequent than A(H1N1)pdm09 in 2014‐2015 (H1N1 10.9%; H3N2 52.0%; B 37.1%), 2016‐2017 (H1N1 0.5%; H3N2 72.9%; B 26.6%), and 2017‐2018 (H1N1 7.0%; H3N2 38.3%; B 54.7%).^1^, ^2^

The difference in clinical manifestation and outcome between A(H1N1)pdm09 and A(H3N2) has not been clearly defined. Before 2009, several observational data analysis described higher influenza‐associated hospitalization rate and mortality in A(H3N2) predominant seasons, but they are generally regarded as insignificant findings.^3^, ^4^ After the H1N1 pandemic in 2009, A(H1N1)pdm09 became a major seasonal strain which was reported to be affected more younger groups, occasionally leading to more serious outcomes than previous strains.^5^, ^6^, ^7^ This result suggests that individuals aged below 30 years lacked the cross‐reactive antibody against A(H1N1)pdm09.^8^ Data in the post‐pandemic era generally showed that A(H1N1)pdm09 was associated with younger patients and greater severity compared to A(H3N2).^9^, ^10^, ^11^, ^12^, ^13^ However, some inconsistencies exist due to heterogeneous setting of each study such as different participated countries, healthcare resources, vaccination rates, and other potential confounders. Therefore, we pursue to reveal more clear evidence of difference between influenza A subtypes with minimizing confounders in highly vaccinated, well‐developed single country, South Korea. The potential discrepancy of influenza burden among subtypes may inform the global influenza policy depending on the seasonal predominant subtype. In this study, we aimed to determine the difference in epidemiology, clinical manifestations and outcomes between patients with A(H1N1)pdm09 infection and those with A(H3N2) infection from 2014 to 2018 in South Korea using data from the influenza surveillance network.

## 2 METHODS

### 2.1 Study population and data collection

From August 31, 2014, to August 25, 2018, nine tertiary care hospitals (Korea University Guro Hospital, Korea University Ansan Hospital, St. Vincent's Hospital of the Catholic University of Korea College of Medicine, Kyungpook National University Hospital, Chonnam National University Hospital, Chungbuk National University Hospital, Yonsei University Wonju Hospital, Inha University Hospital, and Hallym University Kangnam Sacred Heart Hospital) in South Korea participated in the hospital‐based influenza morbidity and mortality (HIMM) surveillance system. The surveillance system is composed of the following two types of schemes: emergency room (ER)‐based and hospitalized patient‐based influenza‐like illness (ILI)/severe acute respiratory infection (SARI) surveillance.

Each season of influenza was divided by 1 year: from August 31, 2014, to August 29, 2015 (2014‐2015 season); from August 30, 2015, to August 27, 2016 (2015‐2016 season); from August 28, 2016, to August 26, 2017 (2016‐2017 season); and from August 27, 2017, to August 25, 2018 (2017‐2018 season). ILI was defined as (a) history or sudden onset of fever ≥38.0°C and (b) presence of ≥1 respiratory symptoms such as cough, sore throat, or rhinorrhea/nasal obstruction which onset within 7 days. SARI was defined (a) history or sudden onset of fever ≥38.0°C; (b) presence of ≥1 respiratory symptoms such as cough, sore throat, or rhinorrhea/nasal obstruction which onset within 7 days; (c) shortness of breath; and (d) admission.^14^, ^15^ All adult patients (≥19 years) with ILI or SARI who visited the emergency room (with or without admission) or admitted to the hospital via outpatient clinic were enrolled when they were agreed to participate in the study. Written informed consent was obtained before the specimen collection. After enrollment, two viral samples were collected via nasopharyngeal swab. One sample was immediately underwent rapid antigen tests (RAT) using BD Veritor System to diagnose influenza infection. Another one sample was transferred to the center hospital, Korea University Guro Hospital, and subsequent subtype identification (A(H1N1)pdm09, A(H3N2) or B) was performed by StepOne^TM^ Real‐Time Polymerase Chain Reaction (PCR) System. When the subtype data were unavailable by fulfilling more than one of the followings: (a) PCR results were negative; (b) sample was inadequate to analyze due to inappropriate storage or missing during transportation; (c) PCR analysis was impossible because of insufficient laboratory capacity; and (d) more than two subtypes of influenza were identified in one sample, we introduced the subtype results from commercial respiratory virus real‐time PCR kit or RAT kits performed in each contributing hospital if possible. The case with unavailable subtype data had been excluded from the analysis.

The clinical data of subtype‐confirmed patients were collected by retrospectively reviewing medical records. Data on demographic characteristics, histories of vaccination, hospitalization, underlying diseases, clinical manifestations, complications, and in‐hospital mortality were collected using a case report form. Pneumonia was defined as respiratory symptoms with pulmonary infiltrations on chest radiography, which was confirmed by a radiologist. All hospitalized patients were followed up to 90 days along the hospital stay and checked the date of death or the date of discharged alive.

The HIMM surveillance including participant enrollment, sample collection, and data analysis was approved by the institutional review board at each contributing hospital and the center hospital, Korea University Guro Hospital (approval nos. KUGH11088 for 2014‐2015, 2015‐2016 and 2016‐2017 seasons, and 2017GR0172 for 2017‐2018 season).

### 2.2 Data management and statistical analysis

All categorical clinical data were calculated and analyzed using chi‐square and Fisher's exact test. Mann‐Whitney's *U* test was used to compare the mean age and length of hospital stay between two groups, and the results were expressed as median (interquartile range). A *P* value of <.05 was considered to significant. Potential variables that might be associated to the development of pneumonia during influenza A infection were analyzed by multivariable linear model logistic regression as adjusted odds ratios (ORs). Variables which were significantly associated with pneumonia in the univariable analysis (*P* < .05) and generally known risk factors of pneumonia (eg, age and underlying diseases) were selected and included in multivariable regression analysis. The association between A(H1N1)pdm09 and A(H3N2) in relation to the overall mortality of hospitalized patients was investigated using a time‐dependent Cox regression analysis to obtain the hazard ratios (HRs). All variables were validated by the graphical analysis of log‐minus‐log plot for the proportional hazards assumption. All statistical analyses were performed using Statistical Package for the Social Sciences version 20.0. The post hoc power analysis of Cox regression was conducted by Power Analysis & Sample Size 2020, NCSS Statistical Software.

## 3 RESULTS

### 3.1 Study population and baseline characteristics

A total of 4760 patients diagnosed with influenza infection were enrolled in the study. A total of 2741 were enrolled at the time of ER visit with 2066 were discharged from ER and 675 were admitted to the wards or ICUs, whereas 2019 were enrolled at wards after admission (Figure S1). At the point of view of the influenza subtype, there were 240 patients of A(H1N1)pdm09, 1507 patients of A(H3N2), and 998 patients of B. In addition, 1974 were influenza A confirmed by RAT but could not obtain subtype results and 41 were more than one subtype detected in single analysis. Of them, 448 cases were negative PCR results, seven cases were inadequate sample due to lost or inappropriate storage, and 1519 cases did not performed PCR because of insufficient laboratory capacity (Table 1). In cases of subtype‐confirmed influenza A infection (n = 1747), a total of 1591 patients were confirmed in a laboratory of center hospital via real‐time PCR, 147 via in‐hospital commercial respiratory virus real‐time PCR kit, and nine via in‐hospital RAT kit. The numbers of participants in each season were as follows: 1749 in the 2014‐2015 season, 1141 in the 2015‐2016 season, 783 in the 2016‐2017 season, and 1087 in the 2017‐2018 season. A(H1N1)pdm09 was dominant in the 2015‐2016 season, while A(H3N2) was dominant in the rest of the seasons.

**TABLE 1:** Seasonal numbers of participants with influenza A subtype and B

	H1N1pdm09 (n = 240)	H3N2 (n = 1507)	B (n = 998)	A with unknown subtype^a^ (n = 1974)	More than one subtype detected^b^ (n = 41)
2014‐2015	33	813	386	509	8
2015‐2016	201	94	189	632	25
2016‐2017	0	270	59	451	3
2017‐2018	6	330	364	382	5

^a^ Including negative PCR results or PCR analyses were not performed due to limited laboratory capacity or inadequate specimen.

^b^ More than one subtype detected in single analysis (eg, H1N1pdm09 and H3N2 were simultaneously detected in single PCR analysis, or A and B were simultaneously detected in single RAT kit).

**FIGURE 1:**
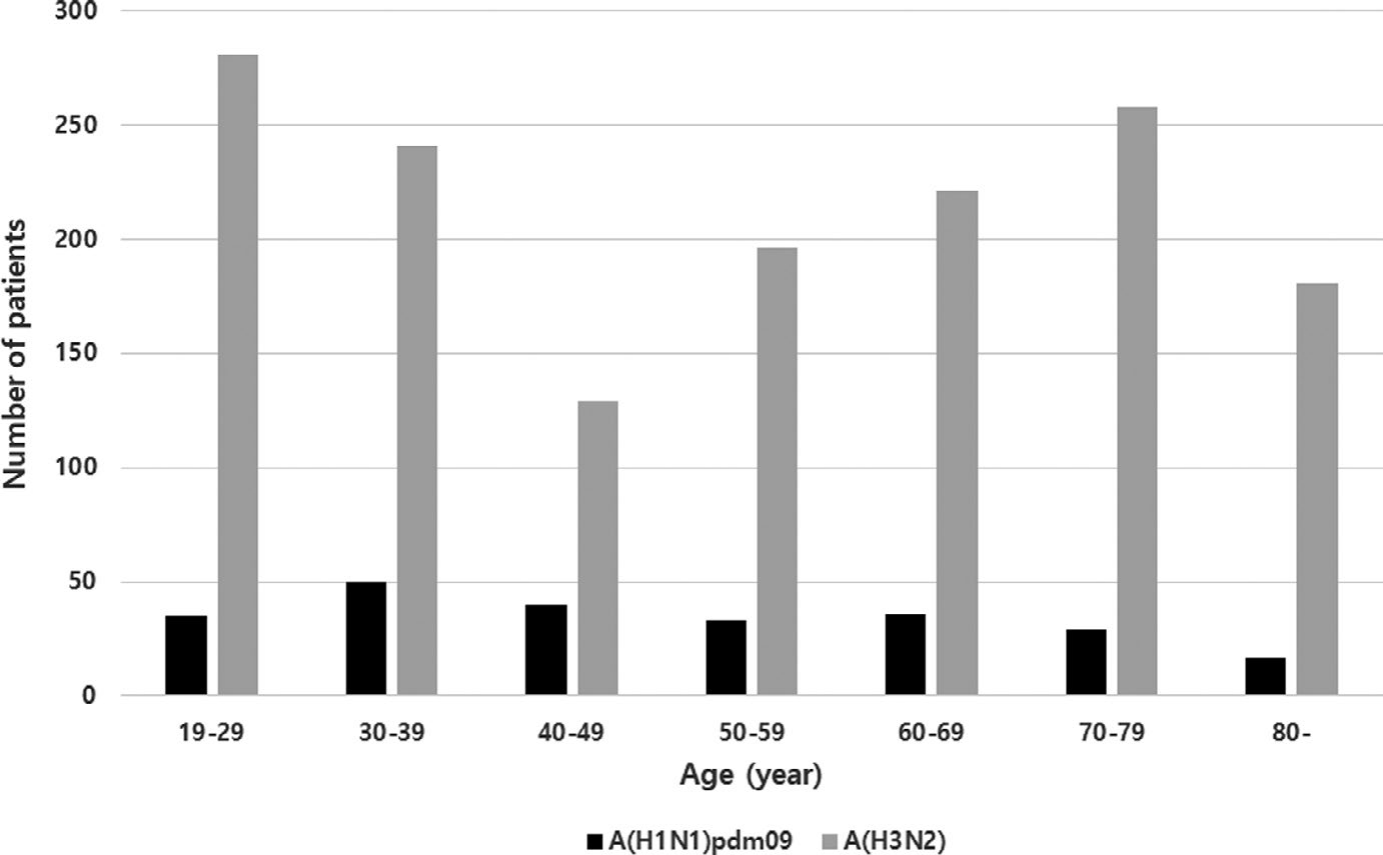
Age group distribution of A(H1N1)pdm09 and A(H3N2) subtypes

The baseline characteristics of the A(H1N1)pdm09 and A(H3N2) groups are shown in Table 2. Mean age was significantly lower in the A(H1N1)pdm09 group than in the A(H3N2) group (50.0 years with standard deviation 18.8 vs 53.4 years with standard deviation 21.1, *P* = .030), and the median age difference was 7 years. The proportions of patients aged 19‐49 years and 50‐64 years were higher in the A(H1N1)pdm09 group (52.1% vs 43.2% and 22.5% vs 19.8%, *P* = .002), while the proportion of patients aged above 65 years was higher in the A(H3N2) group (25.4% vs 37.0%, *P* = .002). Age group distribution by 10 years showed a gradual decrease in the proportion of patients with A(H1N1)pdm09 with aging and bimodal curve of A(H3N2) (Figure 1). Compared with the A(H3N2) group, fewer patients from the A(H1N1)pdm09 group received seasonal influenza and pneumococcal vaccines (27.9% vs 43.9% in influenza vaccination, *P* < .001, 41.0% vs 51.9% in ≥65 years age group in pneumococcal vaccination, *P* < .001). The A(H3N2) group had a higher proportion of patients with more than one underlying disease than the A(H1N1)pdm09 group (67.5% vs 74.0%, *P* = .035). Patients in the A(H1N1)pdm09 group more frequently significantly experienced fever, chills, cough, nausea/vomiting, headache, and wheezing than those in the A(H3N2) group.

**TABLE 2:** Baseline characteristics of patients with subtype‐confirmed influenza A infection

	H1N1pdm09 (n = 240)	H3N2 (n = 1507)	*P* value
Sex (male)(%)	100 (41.7)	614 (40.7)	.787
Age			
Mean ± SD	50.0 ± 18.8	53.4 ± 21.1	.030
Median (IQR)	48 (34.25‐65)	55 (33‐72)	
Age group(%)			
19‐49 y	125 (52.1)	651 (43.2)	.002
50‐64 y	54 (22.5)	299 (19.8)	
≥65 y	61 (25.4)	557 (37.0)	
History of influenza vaccination(%)^a^			
Yes	67 (27.9)	661 (43.9)	<.001
No	167 (69.6)	835 (55.4)	
Unknown	6 (2.5)	11 (0.7)	
History of influenza vaccination (in patients aged ≥65 y) (%)^a^			
Yes	32 (52.5)	406 (72.9)	<.001
No	24 (39.3)	147 (26.4)	
Unknown	5 (8.2)	4 (0.7)	
History of pneumococcal vaccination (in patients aged ≥65 y) (%)			
Yes	25 (41.0)	289 (51.9)	<.001
No	29 (47.5)	264 (47.4)	
Unknown	7 (11.5)	4 (0.7)	
Current smoker(%)	28 (11.7)	145 (9.6)	.325
Underlying illness(%)			
Any underlying disease	162 (67.5)	1115 (74.0)	.035
Current smoker(%)	27 (11.2)	249 (16.5)	.038
Cardiovascular disease	18 (7.5)	121 (8.0)	.778
Cerebrovascular disease	11 (4.6)	89 (5.9)	.413
Neuromuscular disease	1 (0.4)	36 (2.4)	.051
Chronic respiratory disease (except COPD)	4 (1.7)	55 (3.6)	.126
COPD	6 (2.5)	78 (5.2)	.072
Asthma	16 (6.7)	63 (4.2)	.085
TB	8 (3.3)	61 (4.0)	.598
CKD	8 (3.3)	45 (3.0)	.771
Chronic liver disease	5 (2.1)	42 (2.8)	.670
Solid cancer	17 (7.1)	124 (8.2)	.545
Hematologic malignancy	5 (2.1)	12 (0.8)	.072
Autoimmune disease	3 (1.2)	10 (0.7)	.406
Immunosuppressant use	7 (2.9)	30 (2.0)	.355
Pregnancy	10 (4.2)	45 (3.0)	.331
Symptoms(%)			
Fever	238 (99.2)	1444 (95.8)	.009
Chill	153 (63.7)	819 (54.3)	.006
Cough	227 (94.6)	1331 (88.3)	.004
Sputum	163 (67.9)	982 (65.2)	.404
Sore throat	110 (45.8)	701 (46.5)	.844
Rhinorrhea/nasal congestion	138 (57.5)	867 (57.5)	.993
Chest pain	22 (9.2)	120 (8.0)	.526
Dyspnea	47 (19.6)	308 (20.4)	.760
Diarrhea	11 (4.6)	87 (5.8)	.457
Nausea/vomiting	41 (17.1)	184 (12.2)	.036
Abdominal pain	11 (4.6)	81 (5.4)	.610
Headache	97 (40.4)	437 (29.0)	<.001
Myalgia	115 (47.9)	642 (42.6)	.123
Wheezing	13 (5.4)	44 (2.9)	.043
General weakness	87 (36.2)	477 (31.7)	.157
Crackle	5 (2.1)	43 (2.9)	.671
Seizure	0	2 (0.1)	1.00

Abbreviations: CKD, chronic kidney disease; COPD, chronic respiratory pulmonary disease; DM, diabetes mellitus; IQR, interquartile range; SD, standard deviation; TB, tuberculosis.

^a^ Influenza vaccination conducted in current season.

### 3.2 Clinical outcomes

Clinical outcomes of subtype‐confirmed influenza A infection are displayed in Table 3. A total of 53 patients with A(H1N1)pdm09 infection (22.1%) and 338 with A(H3N2) infection (22.4%) were hospitalized, while four patients with A(H1N1)pdm09 infection (1.7%) and 51 patients with A(H3N2) infection (3.4%) were admitted to the intensive care unit. Hospitalized patients were older (mean age 70.0 in hospitalized group vs 48.0 in non‐hospitalized group, *P* < .001), had lower vaccination rate (38.4% vs 62.8%, *P* < .001), and had considerably more underlying diseases (91.8% vs 67.7%, *P* < .001) and lower respiratory symptoms such as sputum (73.1% vs 63.3%, *P* < .001) and dyspnea (42.5% vs 13.9%, *P* < .001) (Table S1). The prevalence of pneumonia was significantly higher in the A(H3N2) group than in the A(H1N1)pdm09 group (6.2% vs 11.1%, *P* = .021). No significant difference was observed in the overall mortality between the two groups (4 deaths (2.7%) vs 22 deaths (1.5%), *P* = .774).

**TABLE 3:** Clinical outcomes of subtype‐confirmed influenza A infection

	H1N1pdm09 (n = 240)	H3N2 (n = 1507)	*P* value
Hospital admission (%)	53 (22.1)	338 (22.4)	.905
Hospital LOS (days)			
Overall	9.2 ± 10.3	10.1 ± 11.3	.550
19‐49 y	3.8 ± 2.0	4.2 ± 2.8	.966
50‐64 y	10.5 ± 7.6	9.0 ± 11.6	.300
ICU admission			
Overall	4 (1.7)	51 (3.4)	.229
19‐49 y	0	2 (0.3)	.00
50‐64 y	0	7 (2.3)	.601
≥65 y	4 (6.6)	42 (7.5)	1.00
Pneumonia (viral and/or bacterial)			
Overall	15 (6.2)	168 (11.1)	0.021
19‐49 y	1 (0.8)	8 (1.2)	1.00
50‐64 y	4 (7.4)	26 (8.7)	1.00
≥65 y	10 (16.4)	134 (24.1)	.179
In‐hospital death			
Overall	4 (1.7)	22 (1.5)	.774
19‐49 y	0	0	‐
50‐64 y	1 (1.9)	4 (1.3)	.566
≥65 y	3 (4.9)	18 (3.2)	.452

Abbreviations: ICU, intensive care unit; LOS, length of stay.

All pneumonia patients (n = 183) were hospitalized in the surveillance. In the analysis of logistic regression, older age was a risk factor for pneumonia (compared to those aged 19‐49 years, adjusted OR (AOR): 6.9 (95% confidence interval (CI): 3.2‐14.8, *P* < .001) in those aged 50‐64 years; AOR: 19.2 (95% CI: 9.2‐40.1, *P* < .001) in those aged ≥65 years) (Table 4). The AORs of sex (compared to female, male AOR: 1.4 (95% CI: 1.0‐1.9), *P* = .070), underlying illness (compared to no underlying illness, AOR: 1.6 (95% CI: 0.9‐3.0), *P* = .115), and vaccination history (compared to not vaccinated, AOR: 0.9 (95% CI: 0.6‐1.3), *P* = .421) were not statistically significant. The influenza A subtype also did not show a meaningful OR (compared to A(H3N2), A(H1N1)pdm09 AOR: 0.7 (95% CI: 0.4‐1.2, *P* = .172).

**TABLE 4:** Risk factors of pneumonia in subtype‐confirmed influenza A infection calculated using logistic regression analysis^a^

	Population	Pneumonia (%)	Adjusted ORs ^b^	95% CI	*P* value
Total	1747	183 (10.5)			
Sex					
Female	1033	87 (8.4)	1 (Reference)		
Male	714	96 (13.4)	1.4	1.0‐1.9	.070
Age					
19‐49 y	776	9 (1.2)	1 (Reference)		
50‐64 y	353	30 (8.5)	6.9	3.2‐14.8	<.001
≥65 y	618	144 (23.3)	19.2	9.2‐40.1	<.001
Subtype					
	240	15 (6.2)	0.7	0.4‐1.2	.172
H3N2	1507	168 (11.1)	1 (Reference)		
Any underlying diseases	1277	169 (13.2)	1.6	0.9‐3.0	.115
No underlying disease	470	14 (3.0)	1 (Reference)		
Influenza vaccination	728	115 (15.8)	0.9	0.6‐1.3	.421
Not vaccinated	1019	68 (6.7)	1 (Reference)		
Pneumococcal vaccination	387	88 (22.7)	1.4	0.9‐2.0	.097
Not vaccinated	1360	95 (7.0)	1 (Reference)		

Abbreviations: CI, confidence interval; OR, odd ratios.

^a^ Hosmer–Lemeshow goodness‐of‐fit test = 0.402.

^b^ Adjusted for all the variables listed in the table.

**FIGURE 2:**
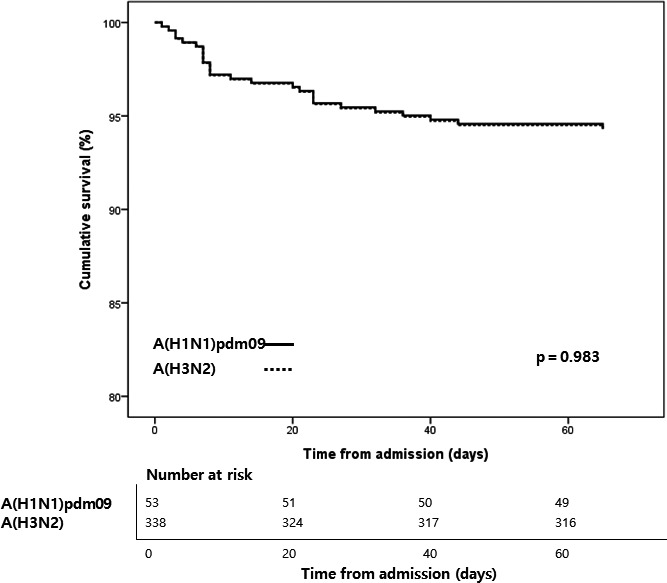
Kaplan‐Meier estimate of in‐hospital mortatlity between H1N1pdm09 and H3N2 among hospitalized patients

**FIGURE 3:**
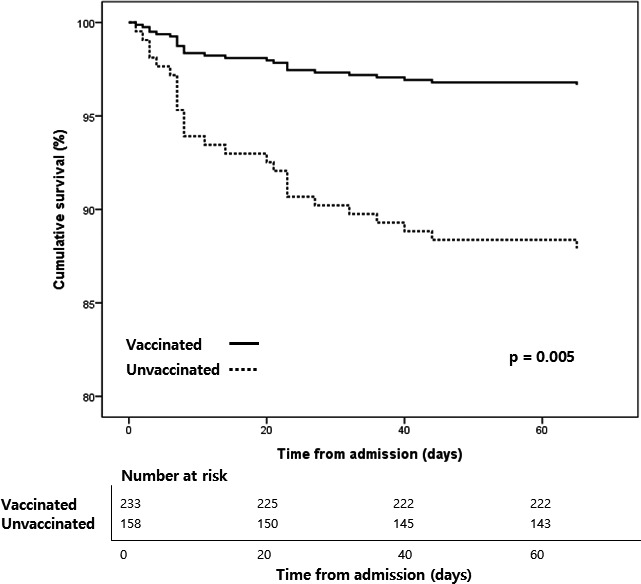
Kaplan‐Meier estimate of in‐hospital mortatlity based on influenza vaccination among hospitalized patients

The HR of variables associated with in‐hospital mortality among hospitalized patients were analyzed by Cox regression (Table 5). The variable of underlying diseases could not be calculated because every hospitalized patient had more than one illness. Participants who received seasonal influenza vaccine showed significantly lower hazards of in‐hospital death (compared to not vaccinated, HR: 0.3 (95% CI: 0.1‐0.7), *P* = .005). Other factors including the influenza A subtype showed no significant difference (Figures 2 and 3).

**TABLE 5:** Risk factors of in‐hospital mortality among hospitalized patients of subtype‐confirmed influenza A infection calculated using Cox regression analysis

	Population	Death (%)	HRs^a^	95% CI	*P* value
Total	391	26 (6.6)			
Sex					
Female	216	13 (6.0)	1 (Reference)		
Male	175	13 (7.4)	1.2	0.6‐2.7	.580
Age					
<65 y	108	5 (4.6)	1 (Reference)		
≥65 y	283	21 (7.4)	2.1	0.7‐5.9	.173
Subtype					
H1N1pdm09	53	4 (7.5)	1.0	0.3‐3.1	.983
H3N2	338	22 (6.5)	1 (Reference)		
Influenza vaccination	233	11 (4.7)	0.3	0.1‐0.7	.005
Not vaccinated	158	15 (9.5)	1 (Reference)		
Pneumococcal vaccination	170	13 (7.6)	2.1	0.8‐5.3	.124
Not vaccinated	221	13 (5.9)	1 (Reference)		

Abbreviations: CI, confidence interval; HR, hazard ratios.

^a^ Adjusted for all the variables listed in the table.

## 4 DISCUSSION

Subtypes of influenza A, A(H1N1)pdm09 and A(H3N2), did not show a significant influence on the development of pneumonia and in‐hospital mortality. Old age, high rate of vaccination, underlying disease, and pneumonia were associated with A(H3N2) infection. However, when the age, sex, underlying disease, and vaccination were adjusted in the logistic regression among hospitalized pneumonia and Cox regression among hospitalized participants, no significant difference was observed between the A(H1N1)pdm09 and A(H3N2) groups.

In 2009, the A(H1N1)pdm09 pandemic strain caused more severe infections in young patients than previous A(H3N2) strains.^5^, ^6^, ^7^ Following this, A(H1N1)pdm09 and A(H3N2) showed an annual global predominance, but the detailed proportion was randomly different. Our study was started 5 years after the 2009 pandemic to determine whether the clinical characteristics of A(H1N1)pdm09 remained the same or had changed. The possible difference between subtypes was expected to offer refined information of the disease burden of influenza A in each season and contribute to the changes in public health policy.

Influenza vaccination rate was higher in the A(H3N2) group than in the A(H1N1)pdm09 group, which possibly indicates that the efficacy of this vaccine against A(H3N2) strain is lower than that against A(H1N1)pdm09 strain. The meta‐analysis from 2004 to 2015 showed poor protection rate of seasonal influenza vaccine against A(H3N2) strain.^16^ Moreover, the high proportion of older adults in the A(H3N2) group might contribute to the high vaccination rate. Indeed, the group aged above 65 years tended to participate in influenza vaccination because South Korea implemented a national group immunization program on this age group and on high‐risk immunocompromised individuals using a trivalent vaccine. The high influenza immunization rate in senior groups might also be associated with a high pneumococcal immunization rate, which was also introduced as a national immunization program in South Korea. Total morbidity and mortality of influenza are generally known to be related to the influenza‐bacterial co‐infection, superinfection of *Streptococcus pneumoniae,* and pneumococcal vaccination.^17^, ^18^ Therefore, influenza vaccination and pneumococcal vaccination were all considered important variables in the analysis.

The high susceptibility of A(H1N1)pdm09 infection among younger patients could be explained by the different possession rate of cross‐reactive antibody and birth cohort effect, which also known as immunologic imprinting caused by lack of previous childhood exposure of similar A(H1N1) viruses.^8^, ^19^, ^20^, ^21^, ^22^ Since the age‐dependent difference of subtype susceptibility could affect to the difference of clinical outcomes, it should also be adjusted in the analysis. Furthermore, patients aged below 65 years received fewer influenza vaccinations than older people because they are not included in the free vaccination program except for the high‐risk group with underlying illness. The influenza vaccination rate in South Korea, as surveyed by the Ministry of Health and Welfare, was 28.2% in individuals aged 19‐64 years and 82.7% in those aged ≥65 years.^23^ However, the incidence of serious influenza infection requiring hospitalization and the mortality rate were lower in younger patients.^24^ All the above factors including age and vaccination history had complex relationships with each other which may confound the study results. We used a logistic regression analysis to compare these factors, and no significant difference was observed in the pneumonia incidence between the A(H1N1)pdm09 and A(H3N2) subtypes. A recent systematic literature review assessed 47 studies and did not observe any significant difference in secondary bacterial pneumonia, ICU admission, and death between the subtypes of influenza A and B.^9^ Statistically insignificant results about subtype difference might be associated with sparse number of participants in the study. However, up to now, results in our study and related researches showed that A(H1N1)pdm09 generally does not seem to be more virulent than A(H3N2).

In the Cox regression analysis, hospitalized patients who received influenza vaccination had low risk of in‐hospital mortality, and the age group, sex, subtypes, and pneumococcal vaccination did not show any significant effect on in‐hospital mortality. High hazards ratio in older age groups (≥65 years) without statistical significance might be associated with insufficient number of participants and deaths in the analysis. The effectiveness of influenza vaccine is well known. However, there is a controversy regarding whether the vaccine can certainly reduce mortality.^25^ Recently, an analysis of high‐risk individuals with chronic obstructive pulmonary disease or heart failure showed a benefit of all‐cause mortality in the influenza vaccinated group.^26^, ^27^ This finding is comparable to our result because all hospitalized participants in our analysis had more than one underlying illness. Moreover, South Korea has a relatively well‐organized immunization program with a high vaccination rate; the impact of vaccination decreasing mortality might be more clearly expressed in our studies than in previous studies in poorly vaccinated countries. This result means that although the seasonal influenza vaccination could not prevent influenza infection and hospitalization, it may help reduce mortality, which supports the importance of seasonal vaccination in older adults and high‐risk individuals with underlying diseases. We conducted the post hoc power analysis of Cox regression which achieves 91% power at a 0.05 significance level to detect a regression coefficient of influenza vaccination.

In addition, we also conducted Cox analysis by subgroup, showed that the difference in HR based on influenza vaccination was only significant in the A(H3N2) group but not in the A(H1N1)pdm09 group (A(H1N1)pdm09 group, HR: 0.3 (95% CI: 0.0‐8.4), *P* = .474; A(H3N2)group, HR: 0.3 (95% CI: 0.1‐0.7), *P* = .006, data not shown in the table). This result might be because of the size of the A(H1N1)pdm09 group but also might be influenced by lack of power and multiple‐comparison bias.

The study has a number of limitations, including its retrospective nature. First, the study populations might have more underlying diseases than the general population because most of the enrolled participants were recruited from the emergency rooms and wards of tertiary care hospitals. It is possible that a considerable number of mild influenza infections treated in outpatient clinics or even unrecognized by clinicians were ignored by the surveillance system. This selection bias might be associated with the incidence of pneumonia and death than the virtual influenza burden. Therefore, conducting a cohort surveillance including patients with mild infections would be helpful in determining the definite characteristics of influenza. Second, prescription of antiviral agents, use of adjunctive glucocorticoids, exact timing and type of pneumococcal vaccine, and influenza vaccination in previous seasons were not included in the analysis, which can be confounders of the study results. Furthermore, differences in regional demography and the treatment strategy used by each clinician from participating hospitals should be considered. Third, we did not perform real‐time PCR as subtype confirmation for all influenza A samples because of limited resources and laboratory capacity. Despite there were no factors influenced whether or not the PCR was conducted except laboratory situation, potential selection bias might have been involved. Finally, the low effectiveness of conventional influenza vaccines in older adults has been reported; hence, it is difficult to determine the direct link between influenza vaccination history and reduction of in‐hospital mortality.^28^ Generally low fatality of influenza virus itself and moderate effectiveness of vaccine might also contribute to reduce the power of analysis. Large‐scale and controlled prospective studies in individuals with severe influenza infections would be necessary to investigate this issue.

In conclusion, there was no significant difference in pneumonia and in‐hospital mortality rates between A(H1N1)pdm09 and A(H3N2) infections when adjusted with age, sex, underlying diseases and vaccination. The younger age group was more susceptible to A(H1N1)pdm09 but had fewer risk of pneumonia development than the older age group. Reduced in‐hospital mortality in vaccinated hospitalized patients suggests importance of seasonal influenza vaccination. Prospective large controlled cohort studies would be necessary to clearly describe the differences in clinical features of the influenza subtypes.

## CONFLICT OF INTEREST

The authors have no potential conflicts of interest to disclose.

## AUTHOR CONTRIBUTION

**Jin Gu Yoon:** Formal analysis (lead); Writing‐original draft (lead). **Ji Yoon Noh:** Conceptualization (supporting); Writing‐review & editing (supporting). **Won Suk Choi:** Resources (equal). **Jacob Lee:** Resources (equal). **Jin Soo Lee:** Resources (equal). **Seong‐Heon Wie:** Resources (equal). **Young Keun Kim:** Resources (equal). **Hye Won Jeong:** Resources (equal). **Shin Woo Kim:** Resources (equal). **Kyung‐Hwa Park:** Resources (equal). **Joon Young Song:** Conceptualization (supporting); Writing‐review & editing (supporting). **Hee Jin Cheong:** Conceptualization (supporting); Writing‐review & editing (supporting). **Woo Joo Kim:** Conceptualization (lead); Writing‐review & editing (lead).

## Supporting information

App S1
