# Supplementary material for: A comparison of epidemiology and clinical outcomes between influenza A H1N1pdm09 and H3N2 based on multicenter surveillance from 2014 to 2018 in South Korea
Source: Influenza Other Respir Viruses. 2020 Aug 25;15(1):99–109. doi: 10.1111/irv.12795 (PMC7767957; doi:10.1111/irv.12795)

**Appendix S1**

**Supplementary Table 1. Baseline characteristics of patients with influenza A infection between hospitalized and non-hospitalized patients**

|  | Hospitalized  (n=391) | Non-hospitalized  (n=1,356) | p value |
| --- | --- | --- | --- |
| Sex (male)(%) | 175 (44.8) | 539 (39.7) | .076 |
| Age  Mean±SD | 70.0±15.5 | 48.0±19.5 | <.001 |
| Median (IQR) | 74 (63-80) | 46 (31-64) |  |
| Age group(%)  19-49years  50-64 years  ≥65 years | 43 (11.0)  65 (16.6)  283 (72.4) | 733 (54.1)  288 (21.2)  335 (24.7) | <.001 |
| History of influenza vaccination(%)  Yes  No  Unknown | 150 (38.4)  233 (59.6)  8 (2.0) | 852 (62.8)  495 (36.5)  9 (0.7) | <.001 |
| History of pneumococcal vaccination (in patients aged ≥65 years) (%)  Yes  No  Unknown | 211 (54.0)  170 (43.5)  10 (2.6) | 1,124 (82.9)  217 (16.0)  15 (1.1) | <.001 |
| Current smoker(%) | 29 (7.4) | 144 (10.6) | .062 |
| Underlying illness(%) |  |  |  |
| Any underlying disease | 359 (91.8) | 918 (67.7) | <.001 |
| DM | 114 (29.2) | 162 (11.9) | <.001 |
| Cardiovascular disease | 66 (16.9) | 73 (5.4) | <.001 |
| Cerebrovascular disease | 44 (11.3) | 56 (4.1) | <.001 |
| Neuromuscular disease | 14 (3.6) | 23 (1.7) | .023 |
| Chronic respiratory disease (except COPD) | 24 (6.1) | 35 (2.6) | .001 |
| COPD | 51 (13.0) | 33 (2.4) | <.001 |
| Asthma | 39 (10.0) | 40 (2.9) | <.001 |
| TB | 36 (9.2) | 33 (2.4) | <.001 |
| CKD | 26 (6.6) | 27 (2.0) | <.001 |
| Chronic liver disease | 11 (2.8) | 36 (2.7) | .865 |
| Solid cancer | 50 (12.8) | 91 (6.7) | <.001 |
| Hematologic malignancy | 10 (2.6) | 7 (0.5) | <.001 |
| Autoimmune disease | 3 (0.8) | 10 (0.7) | .952 |
| Immunosuppressant use | 15 (3.8) | 22 (1.6) | .007 |
| Pregnancy | 7 (1.8) | 48 (3.5) | .081 |
| Symptoms(%) |  |  |  |
| Fever | 340 (87.0) | 1,342 (99.0) | <.001 |
| Chill | 199 (50.9) | 773 (57.0) | .032 |
| Cough | 343 (87.7) | 1,215 (89.6) | .292 |
| Sputum | 286 (73.1) | 859 (63.3) | <.001 |
| Sore throat | 87 (22.3) | 724 (53.4) | <.001 |
| Rhinorrhea/nasal congestion | 156 (39.9) | 849 (62.6) | <.001 |
| Chest pain | 25 (6.4) | 117 (8.6) | .154 |
| Dyspnea | 166 (42.5) | 189 (13.9) | <.001 |
| Diarrhea | 25 (6.4) | 73 (5.4) | .444 |
| Nausea/vomiting | 44 (11.3) | 181 (13.3) | .276 |
| Abdominal pain | 18 (4.6) | 74 (5.5) | .506 |
| Headache | 51 (13.0) | 483 (35.6) | <.001 |
| Myalgia | 93 (23.8) | 664 (49.0) | <.001 |
| Wheezing | 41 (10.5) | 16 (1.2) | <.001 |
| General weakness | 156 (39.9) | 408 (30.1) | <.001 |
| Crackle | 33 (8.4) | 15 (1.1) | <.001 |
| Seizure | 1 (0.3) | 1 (0.1) | .398 |

**Supplementary Figure 1. Flow of study participant enrollment**


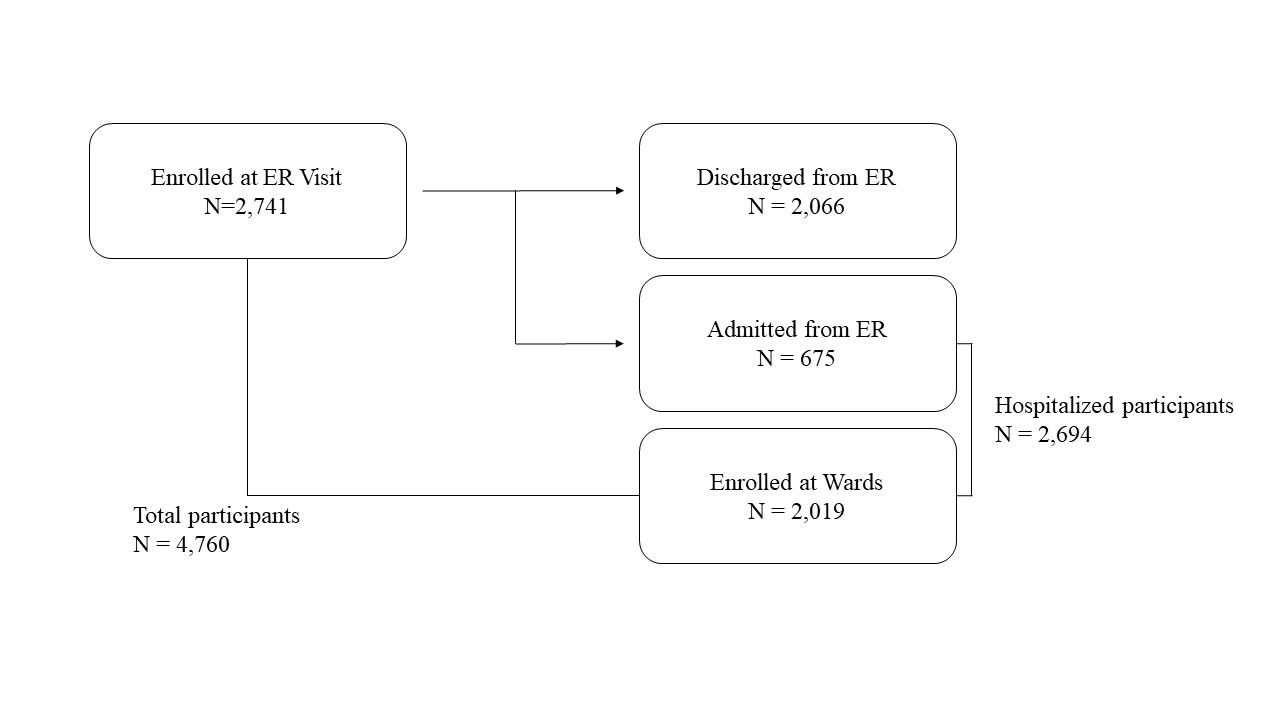

Supplement: Supplementary file 1 — App S1 [file IRV-15-99-s001.docx]
